# Primary Melanoma of the Lung: A Systematic Review

**DOI:** 10.3390/medicina56110576

**Published:** 2020-10-30

**Authors:** Panagiotis Paliogiannis, Antonella M. Fara, Gianfranco Pintus, Wael M. Abdel-Rahman, Maria Colombino, Milena Casula, Giuseppe Palmieri, Antonio Cossu

**Affiliations:** 1 Department of Medical, Surgical and Experimental Surgery, University of Sassari, Viale San Pietro 43, 07100 Sassari, Italy; antonellam.fara@tiscali.it (A.M.F.); cossu@uniss.it (A.C.); 2 Department of Medical Laboratory Sciences, College of Health Sciences, and Sharjah Institute for Medical Research, University of Sharjah, University City Rd, Sharjah 27272, UAE; gpintus@sharjah.ac.ae (G.P.); whassan@sharjah.ac.ae (W.M.A.-R.); 3 Department of Biomedical Sciences, University of Sassari, Viale San Pietro, 07100 Sassari, Italy; 4 Institute of Genetic and Biomedical Research (IRGB) of Sassari, National Research Council, Traversa La Crucca 3, 07100 Sassari, Italy; colombinom@yahoo.it (M.C.); casulam@yahoo.it (M.C.); gpalmieri@yahoo.com (G.P.)

**Keywords:** cancer, malignant melanoma, lung, melan-A, HMB, BRAF

## Abstract

*Background and Objectives*: The respiratory apparatus, generally affected by highly aggressive tumors like lung cancer and mesothelioma, is rarely affected by primary malignant melanoma. The aim of this review was to identify cases of primary malignant melanoma of the lung (PMML) published in the modern scientific literature, and to describe their main clinical, pathological and therapeutic features. *Materials and Methods*: A systematic search of publications in the electronic database PubMed has been performed using keywords, and the references of the selected articles were checked to identify additional missing studies. *Results*: Globally 52 papers reporting on 76 cases were identified. Among them there were 47 reports of a single case, three papers reporting on two cases each, and two larger case series published in 1997 and 2005 including eight and 15 cases, respectively. *Conclusions*: PMML was generally diagnosed in middle-aged males, without any apparent correlation with cigarette smoking. It was more frequently found in the lower lobes and the left lung. The tumors were generally pigmented, composed by epithelial and/or spindle cells with large nuclei and prominent nucleoli, nuclear atypia, and numerous mitotic figures; they commonly showed immunostaining for S-100, HMB 45 and Melan-A. Early detection and surgical resection were the main determinants of survival from this rare malignancy.

## 1. Introduction

Malignant melanoma (MM) is one of the most common malignancies of the skin, but it can potentially affect all areas of the human body [1]. The respiratory apparatus, generally affected by highly incident and prognostically aggressive tumors like lung cancer and mesothelioma, is however rarely affected by primary MM [2,3]. Indeed, only few cases of primary MM of the lung (PMML) in the form of case reports and case series have been described in the scientific literature so far. As a consequence, the pathological features of these melanomas, their incidence, their clinical behavior and the possible therapeutic options to adopt, are not well-established. Furthermore, some of the cases have been published more than 50 years ago and were managed with older diagnostic and therapeutic approaches. Finally, to date, no systematic reviews of the reported cases have been performed. For these reasons, we performed a systematic review of the cases published in the PubMed database in the last 30 years, with the aim of accurately putting together all the current knowledge regarding this rare malignancy.

## 2. Materials and Methods

### 2.1. Search Strategy, Eligibility Criteria and Study Selection

A systematic search of publications in the electronic database PubMed has been performed using the following keywords: (((primary) AND (lung)) AND (malignant)) AND (melanoma). Abstracts were screened, and the selected full-text articles were independently reviewed by two investigators (AMF and PP) to establish relevance. Criteria for article enrollment were: (a) articles reporting cases of pathologically diagnosed primary lung melanomas; (b) articles including demographic, anthropometric, clinical, pathological and treatment details; (c) articles written in English; (d) articles published between 1st January 1990 and 31 December 2019. References of the retrieved articles were also cross-checked to identify additional missing studies. Any disagreement between the reviewers was resolved by a third pathologist (AC). The following information was extracted from each paper and entered in an MS Excel spread-sheet: article title, first author, year of publication, number of cases, age, sex, clinical and family history of the patients, anatomic localization, pathology and/or molecular features of the tumors, treatments adopted and survival times.

### 2.2. Statistical Analysis and Reporting

A descriptive statistical analysis of the findings was performed, using means and standard deviations, medians and interquartile ranges (IQR), absolute ranges and percentages. Differences in survival between patients who underwent surgery and those who did not were performed with the Mann-Whitney test. Statistical analyses were performed using MedCalc for MS Windows, version 15.4 64 bit (MedCalc Software, Ostend, Belgium). The results were reported in accordance with the PRISMA guidelines for systematic reviews and metanalyses.

## 3. Results

**Figure 1:**
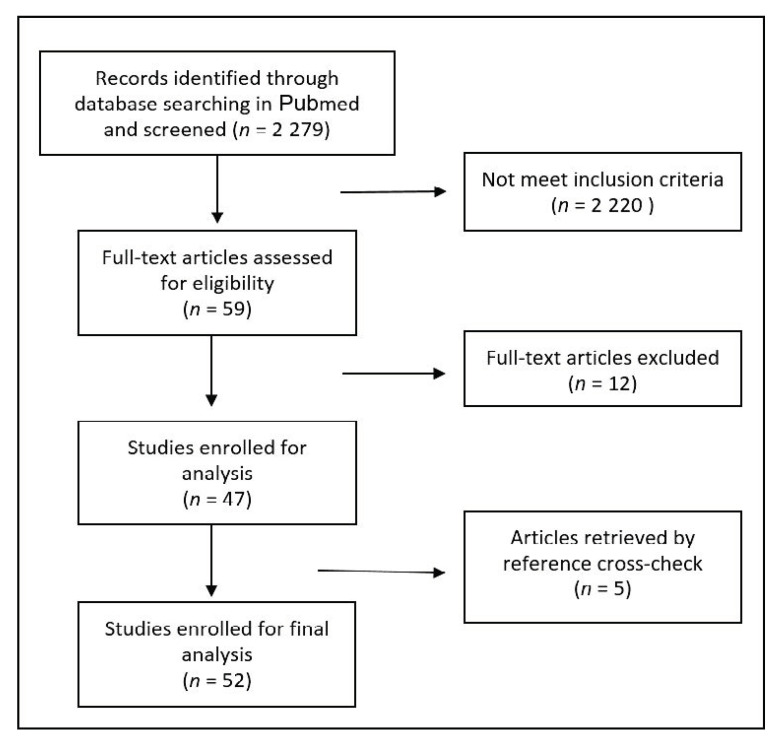
Flowchart illustrating the article selection process.

A flow chart describing the screening process is depicted in Figure 1. There were no disagreements between the reviewers in selecting the articles to enroll; globally 52 papers reporting on 76 cases were selected [4,5,6,7,8,9,10,11,12,13,14,15,16,17,18,19,20,21,22,23,24,25,26,27,28,29,30,31,32,33,34,35,36,37,38,39,40,41,42,43,44,45,46,47,48,49,50,51,52,53,54,55]. Among them there were 47 reports of a single case, three papers reporting on two cases each, and two larger case series published in 1997 and 2005 including eight and 15 cases, respectively.

The median (IQR) age of the 76 patients analyzed was 60 (51.25–68) years and among them 47 (64.4%) were males. The ethnic origin of the patients was reported in 22 cases: 13 (59.1%) were Caucasian, eight (36.4%) were Asian and one (4.5%) African. Data about the family history of cancer were available in eight papers and among them cancer in the patient’s immediate family was reported in two (25%) cases. In addition, data on personal history of cancer were available in 17 cases, ten (58.8%) without any history of malignancy, and seven (41.2%) with at least one previous tumor. Concomitant respiratory diseases were detected in three (27.3%) out of the eleven patients with available data. Regarding smoking, there were eleven (33.3%) active smokers, seven (21.2%) ex-smokers, and 15 (45.5%) never smokers included in the 33 cases with available data. Moreover, data regarding previously excised skin lesions were available in 60 cases; among them only five (8.3%) patients had a history of an excised skin lesion, but not related to the subsequently diagnosed pulmonary melanoma.

The clinical manifestations of the disease were reported in all the cases. The most common signs and symptoms were related to metastasis (41 cases, 53.9%), cough (24 cases, 31.8%), hemoptysis (12 cases, 15.8%), dyspnea (10 cases, 13.1%), chest pain (nine cases, 11.8%), weight loss (eight cases, 10.5%), and fever (four cases, 5.3%). Other rarer manifestations were reported in 26 (34.2%) cases, while the cases completely asymptomatic were seven (9.2%).

The anatomical site of the lesions was reported in 71 cases. The left lung was affected in 35 (49.3%) cases and the right lung in 30 (42.2%), while in six (8.4%) cases the diseases was extended to both lungs at the time of diagnosis. Among the cases with information regarding the pulmonary lobes involved (including cases with both lungs involved), the lower, upper and median lobes were affected in 36 (48%), 31 (41.3%), and 8 (10.6%) cases, respectively. Only in 31 lesions was it reported whether they were located in a central (22, 71%) or peripheral (9, 29%) area of the affected lung, and in 25 cases the presence (8, 32%) or absence (17, 68%) mediastinal lymph node involvement. Data regarding distant visceral metastases were available in 55 cases; 37 (67.3%) of the patients had at least one distant visceral metastasis at the time of diagnosis. Interestingly, in 14 cases extra-thoracic lymph node involvement has been reported, and in seven (50%) out of them it represented the only distant metastasis site.

Regarding the radiological appearance of the tumors, 57 (80.3%) of those with available data were single nodules or masses, while 14 (19.3%) contained multiple or diffuse lesions; in two cases the lesions were not evidenced radiologically. Distant metastases and pleural effusion were detected by imaging in 24 and eight cases, respectively. Histology was employed for pathological diagnosis in all the 73 (100%) cases with available data, while previous cytology was reported in seven (9.6%) cases. Gross descriptions of the tumors were available in 23 cases and among them 20 (86.9%) lesions were described as black nodules or nodules containing colored areas and 11 (47.8%) of them have been described as encapsulated nodules. The mean (SD) maximum diameter of the tumors (in cases of multiple tumors the largest tumor was considered) was 5.1 (±2.4) cm. Regarding microscopy, there were two cases where no detailed information was provided. Among the remaining cases pathological descriptions were variably made, and the most common morphological features mentioned were: cytoplasmic pigment (44 mentions), large nuclei (38 mentions), nuclear atypia (36 mentions), prominent nucleoli (30 mentions), mitotic figures (32 mentions), necrosis (29 mentions), epithelioid cells (25 mentions), spindle cells (16 mentions), and ulceration (10 mentions). The immunohistochemical features of the tumors are summarized in Table 1. Nine tumors were tested for *BRAF* mutations, and only one (11.1%) case harboring such mutations was detected. Only one case was tested for *NRAS* mutations and it was found to be wild type.

Surgery, when feasible, was the main option for the treatment of PMML. Data regarding the treatment options adopted were available in 72 cases, and among them 51 (70.8%) had 56 surgeries (in five cases the patients underwent surgery twice, Table 2). The most common surgical procedures performed were lobectomies and wedge parenchymal resections (Table 2). 

Chemotherapy was used in 20 cases; Dacarbazine alone was the treatment most frequently reported. Combined treatments, some of them including dacarbazine were reported in a few reports. Immunotherapy (with either nivolumab or ipilimumab) was reported in eight recent cases, and targeted therapy in the single *BRAF*-mutated case (Table 2).

The mean survival time was 24.3 (SD: 28.3; median: 13; 95% CI for the median: 7–19; IQR: 5–32) months. The mean overall survival calculated including the 36 deceased patients was 14.3 months (SD: 17.5; median: 6; 95% CI for the median: 4.6–14.4; IQR: 3.5–18.5) months. The median survival was significantly higher in patients who underwent surgery in comparison to those who did not (median 18.5, IQR: 7–46 vs. median 4, IQR: 2–6; *p* = 0,0001).

## 4. Discussion

The search strategy used in our study allowed the identification of 52 papers reporting on 76 cases. Rodriguez et al. in a case report published on April 2019 reported that only 41 PMML had been published since 1916, but our systematic search produced a consistently higher number of cases published in the last 30 years, which allows a first global evaluation of the main clinical, pathological and therapeutic issues of the disease. Neri et al. [35] reported on two cases, but only one of them was PMML. Christopoulos et al. published a case of a stage IV malignant melanoma, with a pulmonary mass and cervical lymph node involvement [56]. Pathological analyses performed on the cervical lymph nodes excised led to diagnosis of melanoma, and the authors claimed that the primary tumor was a MM of unknown origin. Therefore, we excluded this case, even if its description perfectly fits with that of a PMML. Rao et al. published a case of a large congenital melanocytic nevus with metastatic melanoma with a probable primary in the lung, but this origin was not ascertained, and thus we did not include the case [57]. A case initially diagnosed as PMML and subsequently revealed to be a metastatic tumor, as described by Shimmyo et al., was also excluded. Conversely, we included the case described by Karpathiou et al. because it is a very likely a PMML, rather than a recurrence occurred 40 years after the primary diagnosis, as supposed by the authors [14]. The largest series included in the study were published by De Wilt et al. in 2005 including 15 cases [54], and Wilson and Moran in 1997 including eight cases [55].

Like its cutaneous counterpart, PMML in our study was found to affect middle-aged adults (median age 60 years), as opposed to lung cancer which generally affects older individuals [58,59,60]. Furthermore, it mostly affects males than females, similarly to cutaneous melanoma and lung cancer in developing countries; in western countries differences in incidence rates of lung cancer between males and females are continuously narrowing due to changes in smoking patterns between sexes [2,60]. Approximately, two thirds of the cases included in our study affected Caucasian patients, and one third Asian patients, with only one case described in Africa; it is not clear if the difference depends merely on the real incidence of the disease, which in its cutaneous form is known to affect more frequently fair-skinned than dark-skinned individuals, or on differences regarding the existence of cancer registries and scientific publishing rates. In addition, it is not clear if sun exposure, a well- known risk factor for the development of cutaneous MM, plays any role in the onset of PMML. What seems to be related with the occurrence of PMML is a personal or family history of cancer, that was encountered respectively in 25% and 41.2% of the cases with available data. Approximately half of the patients with data regarding their smoking habits were never smokers, which suggests a different pathogenic role of cigarette smoking on PMML in comparison to lung cancer. Finally, only in rare cases previous non-malignant cutaneous lesions were excised in PMML patients, suggesting also potential differences with the traditional pathogenic mechanisms of cutaneous MM.

The clinical manifestations of the disease were similar to those of other thoracic malignancies, including cough, hemoptysis, dyspnea, chest pain, and signs and symptoms related to metastases. An interesting case of dark sputum was reported by Fillipini et al., which represents certainly a particular but rare clinical manifestation of PMML [20].

Most of the lesions in our study affected the left lung and showed a predilection for the lower lobes. This trend is different in comparison with lung cancer, which is more prone to affect the right lung and the upper lobes [2]. In addition, 71% of the lesions with available data were resided in the central areas of the lungs, as opposed to lung adenocarcinoma which is currently the most common type of lung cancer and occurs mainly in the peripheral areas of the lungs; these patterns should be kept in mind for differential diagnosis, when clinical elements suggest a PMML. Furthermore, despite 60% of the 55 patients with available data had metastasis at the time of diagnosis, only one third presented mediastinal lymph node involvement; this percentage appears lower than that of cases with lung cancer, where the lymph nodes of the mediastinum are early involved in the metastatic process. On the other hand, extra-thoracic lymph node involvement was reported in 14 cases, and in seven of them it represented the only distant metastasis detected at the time of diagnosis. These figures suggest a different behavior in the lymphatic progression of PMML in comparison with lung cancer, but more well-documented cases are necessary to better establish it.

Cytology was performed in a few cases prior to histology, and it seems to have a role in rise the suspicion of the disease, rather than in establishing the diagnosis. Histological examination was performed in all the cases, and in approximately 87% black nodules or lesions containing pigmented areas were macroscopically described; depigmentation may occur in PMML of advanced less differentiated stages, making the diagnosis particularly difficult, as described by Karpathiou et al. [14]. The mean maximum diameter of the main lesions was approximately 5 cm, which explains the low rate of asymptomatic cases and the consistent percentage of metastases found. The lesions were generally characterized by organoid and less frequently fascicular growth pattern and include epithelioid to spindle cells with a variable percentage and predominance. Other than the presence of other typical morphological features of MM like the presence of cytoplasmic pigment, large nuclei with prominent nucleoli, nuclear atypia, and mitotic figures, were commonly described. Additional features were necrosis, as well as presence of epithelial and to a lesser extend spindle cells. These characteristics, along with immunohistochemical positivity for S-100, HMB 45 and melan-A, and negativity for cytokeratin and chromogranin, are generally enough to reach pathological diagnosis.

Molecular analysis for *BRAF* mutations was performed only in few cases, but it can be explained by the fact that these tests were introduced in clinical practice only recently, as they represent predicting biomarkers of response to therapy with modern targeted agents like dabrafenib or vemurafenib [61]. *BRAF* mutations have been detected in about 50% of cutaneous MM cases and to a lesser extent in other melanoma subtypes [62]. Only one among the eleven cases tested in this series has been found mutated (11.1%), and no conclusions can be drawn regarding the incidence of *BRAF* mutations in PMML, given the small number of cases. Only one wild type *NRAS* case was also reported.

Surgery, when feasible, was the main option for the treatment of PMML, performed in more than 70% of the patients, and in some cases to treat also recurrences. This is a high percentage, in comparison for example with patients with primary lung cancer or malignant mesothelioma [2,3], but it must be kept in mind that in the 15 cases reported by De Wilt et al. and the eight cases reported by Wilson and Moran surgery substantially was one of the selection criteria. The most common surgical procedures performed were lobectomies and wedge parenchymal resections (Table 2). A few data were available regarding chemotherapy which was used in 20 cases; dacarbazine alone was the treatment most frequently reported, mainly in recent cases. Combined treatments, some of them including dacarbazine were reported in a few reports, while immunotherapy (often combined with interferon) was reported in eight recent cases, and targeted therapy in the single *BRAF*-mutated case (Table 2).

The mean survival time reported was 24.3 months, but as we mentioned before, it must be kept in mind that in reports including numerous cases, there were only surgical, and thus, relatively early stage tumors. The mean overall survival calculated including the 36 dead patients was 14.3 months; this figure is even worse than those of lung cancer or malignant mesothelioma in their early stages or locally advanced stages. As expected, the median survival was significantly higher in patients who underwent surgery in comparison to those who did not. The advent of targeted therapies and immunotherapies, which brought consistent advantages in the clinical management of cutaneous MMs [62] and were used in a few recent cases of PMML, may help improving also the outcomes of this rare disease.

Our review has some limitations regarding the diagnosis, clinical evaluation and treatment of PMML, which need to be highlighted. First of all, the criteria for the diagnosis were variable, with the common denominator of a malignant melanoma of the lung with no evidence of other malignancies; nevertheless, the exclusion of other malignancies might not have been highly accurate, especially in an era in which positron emission tomography (PET) and high definition imaging techniques were not widely available. In addition, the precise genetic landscape of these tumors has not been thoroughly investigated; *BRAF* or *NRAS* mutations were rarely searched, while wider sequencing approaches have not been used to describe the genetic alterations that commonly occur in PMML. This would be particularly useful for the exact diagnosis of these tumors and for a modern therapeutic approach based on precision oncology treatments.

## 5. Conclusions

PMML is a very rare disease, with 76 cases described in the last three decades. It generally affects middle-aged adults, especially males, without apparent correlation with cigarette smoking. PMML is more frequently found in the lower lobes and the left lung, with low rates of mediastinal metastases, even when distant metastases are established. The tumors generally formed of pigmented masses of cell with large nuclei and prominent nucleoli, nuclear atypia, and numerous mitotic figures; they commonly show immunostaining for S-100, HMB 45 and melan-A. Approximately 70% of the patients included in the study underwent surgery and had a significantly higher survival time in comparison with those who were treated with chemotherapy or other treatments alone. Several issues regarding the exact diagnosis, molecular characterization and therapy off these rare tumors need to be better elucidated in future studies.

## Figures and Tables

**Table 1:** Immunohistochemical features of the tumors included in the study.

Marker	Cases Tested, *n*	Positive Staining, *n* (%)	Negative Staining, *n* (%)
S-100	55	51 (92.7)	4 (7.3)
HMB 45	53	50 (94.3)	3 (5.7)
Cytokeratin	38	0 (0)	38 (100)
Melan-A	21	18 (85.7)	3 (14.3)
Chromorganin	17	0 (0)	17 (100)
CAM 5.2	13	1 (7.7)	12 (92.3)
AE1/AE3	12	1 (8.3)	11 (91.7)
Vimentin	11	9 (81.8)	2 (18.2)
CEA	10	3 (30)	7 (70)
NSE	5	1 (20)	4 (80)

**Table 2:** Treatment options in patients with primary lung melanoma.

Treatment	No (%)
**Surgery (case with available data)**	**72**
Total patients who underwent surgery	51 (70.8)
**Total surgical procedures**	**56**
Lobectomy	35 (62.5)
Wedge resection	11 (19.6)
Pneumonectomy	7 (12.5)
Broncoscopic resection	2 (3.6)
Other/NA	1 (1.8)
**Chemotherapy (cases with available data)**	**34**
Total patients treated	20 (58.8)
Dacarbazine	5 (25)
Dacarbazine, vincristine, nimustine	3 (15)
Carboplatin	1 (5)
Paclitaxel	1 (5)
Vindesine, temozolomide, dacarbazine, carboplatin	1 (5)
Other/NA	1 (5)
**Radiotherapy**	**8**
**Other therapies**	**14**
Immunotherapy	5 (35.7)
Interferon	2 (14.3)
Interferon + immunotherapy	2 (14.3)
Supportive care	3 (21.4)
Targeted therapy + immunotherapy	1 (7.1)
Other/NA	1 (7.1)
